# 

**Published:** 1870-10

**Authors:** 


### BOOKS AND PERIODICALS.

---

THE HERALD OF HEALTH, published in New York, by Wood & Holbrook, at $2.00 per annum, is a very superior health journal.

---

FIGARO.—A weekly journal, devoted to Music, the Drama and Freemasonry, is published in New York at $3.00 a year, by Hudson & Merret. Persons interested in the subjects mentioned will find a good exponent in Figaro.

---

GOOD HEALTH.—A monthly journal of physical and mental Culture, published by Alex. Moore, 11 Bromfield St., Boston. Price $2.00 a year. It contains the best written articles upon health matters that we have ever read.

---

THE LITTLE CORPORAL is the most popular magazine for Boys and Girls. It has by far the largest circulation of any juvenile magazine in the world, as it deserves to have. Sewell & Miller Pub., Chicago, at only one dollar and a half a year.

---

ZELL'S ENCYCLOPEDIA.—This very excellent work has reached its 40th number. It exhibits a world of care and labor in its publication, and will be by far, the best universal dictionary in existence, when completed. T. Elwood Zell publisher, 17 & 19 S. 6th St. Phila., or John Heath, Genl. Agent at 48 Court St. Binghamton, N. Y., will supply orders by mail.

---

PETERSON'S MAGAZINE.—This very excellent Ladies' Magazine is full of information for the ladies. The September number contains a beautiful engraving called "Kathleen," several excellent stories and any quantity of fashion plates and patterns, all for $2. 00 a year. Chas. J. Peterson, 306 Chestnut St. Philadelphia, Publisher.

---

EVERY SATURDAY, we regard as the best pictorial paper published in America. It is printed upon beautifully tinted paper, the illustrations are original and well executed, and none are copied from European pictorials. The last number contains many excellent illustrations of the Franco-Prussian war as well as several beautiful art pictures. Its literary contents are able and interesting, being contributed by an able corps of Editors and Correspondents.

Price $4. 00 a year. Fields, Osgood & Co., Pub., Boston.

MRS. O. R. L.—*Great Bend, Pa.*—You forgot to inclose a stamp to prepay postage upon the answer demanded. We have any quantity of opportunities to buy postage stamps for other people, but we have found it so expensive that we have entirely abandoned the practice.

J. J.—*Huntington, N. Y.*—The poke root will not answer in the case to which you refer. Better have your friend consult some surgeon,

DR. B. F. C.—*Midway, Ala.*—Your communication came too late for present number of BISTOURY. We will attend to M. Wagner & Co. in our next.

DR. H. M.—*Springfield, Ill.*—Send your drawings to Geo. Tiemann & Co., 67 Chatham street, N. Y. They will make your instruments for you.

J. R. M.—*Phila., Pa.*—Wash your gilt frames in the water in which onions have been boiled, for the removal of "fly spots."

DR. B.—*Coudersport, Pa.*—The book mentioned can be had of Wm. Wood & Co., N. Y.; price, $7. If medical book publishers would advertise their works as they should, we would be saved replying to many such questions.

MRS. R. L.—*Williamsport, Pa.*—We have examined the "McLean & Hooper" sewing machine, and find it to be as good as any, and superior to many, in the market.

DR. E. L.—*Little Meadows, Va.*—Dr. Gross' work on Surgery is what you require. Blanchard & Lee, Pubs. Have forgotten price.

DR. H.—*Wellsville, N. Y.*—Stellway on the Eye. Wm. Wood & Co., N. Y. Price about $7.

DR. H. D. W.—*Venice, N. Y.*—"The Craig Microscope" will be of no value to you in the detection of urinary deposits. Write Jas. W. Queen, 924 Chestnut street, Phila., for his illustrated pamphlet of microscopes.

DRUGGIST—*Palmyra, Mo.*—One drachm of white arsenic dissolved in a pint of water, and sweetened with honey or sugar, will make an excellent "fly poison." Your paper may be saturated with it, dried, and then used as "fly paper" ordinarily is.

DR. M.—*Columbus, Miss.*—Send to Wm. Wood & Co. N. Y., for his catalogue of Medical publications. You will find what you desire there.

J. S. L.—*Hornellsville, N. Y.*—Dr. W. M. Gregg, a practical chemist of this city, can answer your question better than we can.

DR. R. S.—*Port Jervis, N. Y.*—An Atomizer complete will cost you from six to seven dollars. Write to Geo. Tiemann & Co., 67 Chatham street, N. Y.

Experiments at the Botanic Garden, London, showed that a leaf of the *Victoria Regia* would sustain a weight of 426 pounds before sinking. The leaf selected for the trial is said to have been one of the smaller ones of the plant.

THE ELMIRA
EYE AND EAR INSTITUTE,

For the Treatment of all Diseases of the Eye and Ear, and operations in GENERAL SURGERY.

OPEN FROM 8 A. M. TO 5 P. M.

THAD S. UP DE GRAFF, M. D.,

SURGEON.

### Report of Cases,

Under treatment in the ELMIRA EYE AND EAR INSTITUTE, AND THE CITY HOSPITAL, for the Quarter ending Oct., 1870:

| | |
|---|---|
| Under treatment for Granular Lid | 5 |
| " " " Scrofulous Ophthalmia, | 4 |
| " " " Gonorrhœal " | 1 |
| " " " Syphilitic Iritis, | 1 |
| " " " Pannus, | 5 |
| " " " Rheumatic Ophthalmia, | 1 |
| " " " Glaucoma, | 3 |
| " " " Catarrhal Ophthalmia | 6 |
| " " " Simple Conjunctivitis, | 7 |
| " " " Suppurative Keratitis, | 4 |
| " " " Meibomitis | 2 |
| " " " Retinitis | 2 |
| " " " Corneal Staphyloma | 4 |
| " " " Pustular Keratitis | 3 |
| " " " Opacity of Cornea, | 4 |
| " " " Incised wound of Cornea, | 2 |
| " " " Otorrhœa, | 4 |
| " " " Deafness, | 3 |
| " " " Catarrh and Disease of Throat | 10 |
| " " " Spermatorrhœa, | 2 |
| " " " Tertiary Syphilis | 2 |
| " " " Fracture of fore-arm | 1 |
| " " " Fracture of leg | 1 |
| Operated upon for Cataract, | 3 |
| " " " Iridectomy, | 2 |
| " " " Corneal Staphyloma | 2 |
| " " " Entropium, | 4 |
| " " " Extirpation of Eye, | 2 |
| " " " Pterygium, | 2 |
| " " " Strabismus, (Cross Eye,) | 4 |
| " " " Obstruction Lachrymal Duct, | 1 |
| " " " Trichiasis | 2 |
| " " " Hernia of Iris | 1 |
| " " " Hæmorrhoids | 2 |
| " " " Nasal Polypii, | 1 |
| " " " Cancer of Lip, | 1 |
| " " " Club Foot, (Talipes Varus), | 1 |
| " " " Hydrocele, | 1 |
| " " " Aural Polypii, | 1 |
| " " " Encysted tumor | 2 |
| " " " Cleft Palate | 2 |
| " " " Removal of Parotid Gland | 1 |
| " " " Fistula in ano | 1 |
| " " " Curvature of Spine | 1 |
| Insertion of Artificial Eyes, | 4 |
| Minor Surgical Operations, | 10 |
| Total number of Cases, | 124 |

THEO. N. LESCHER, Registrar.

### PREMIUMS.

WEBSTER'S DICTIONARIES.

We will cause to be forwarded, direct from the Publishers, by Express, one copy of "Webster's Unabridged Dictionaries," (price $12.00) 1840 pages, 3,000 illustrations, for *fifty* subscribers to the BISTOURY. Send us $25, with the addresses in full of fifty subscribers, and we will furnish the BISTOURY for one year, to every address, giving the dictionary as a premium, to the getter up of the Club.

For *twenty-four* subscribers at fifty cents each, we will give as a premium, one copy of "Webster's National Pictorial Dictionary," (price $6.00) 1040 pages, and 600 illustrations.

It will be seen that we offer nothing but first-class premiums to those who work for our Magazine. We hope in this manner to double our circulation for volume VII. Send on the subscribers and secure the premiums offered.

### TO THE LADIES.

We are happy to announce that we have made arrangements with Lang, Russell & Co., of this city, to offer their new and celebrated Sewing Machine, the

**"McLean & Hooper,"**

as a premium for every lady, or other person, who will send us

**One Hundred Subscribers to the Bistoury!**

Only think of that! For fifty dollars we will forward one hundred copies of the Bistoury, and an elegant sewing machine, *which we will guarantee to be equal, if not superior, to any in the market.*

The "McLEAN & HOOPER" makes the "Elastic Lock Stitch," which will not rip, even if every third stitch be cut, and takes its thread directly from the spools—no shuttle being used. It will stitch, hem, fell, tuck, quilt, cord, bind, baste, braid, embroider and gather better than any machine in the market. As before stated, we will guarantee it to be a first class machine in every particular.

We have had agents secure us one hundred subscribers in one single day. Our Magazine is cheap, useful, and a great help and convenience in any family. Send for sample Copies—we will furnish them *free* to all who desire to canvass for it. Twenty-eight pages of reading matter in every number.—Only fifty cents a year. Let us see who wins the first sewing machine. Upon the receipt of fifty dollars we will ship, to any address, nicely boxed and packed, a "McLean & Hooper" family sewing machine, with extra needles, oil, oil can, screw-driver, gauge, and printed directions complete. Be careful to give the Name, Post Office, County and State of every subscriber, plainly written. Send the money by draft, or post office order, to

THAD S. UPDEGRAFF, M. D.,
EDITOR OF BISTOURY,
ELMIRA, N. Y.

### THE BISTOURY GIVEN AWAY.

OUR TERMS FOR CLUBBING WITH OTHER JOURNALS.

We will cause any of the publications below to be sent, together with THE BISTOURY, for one year, on the following terms:—

| | Pub, Price. | With Bistoury. |
|---|---|---|
| Good Health, | $ 2.00 | $ 200 |
| Peterson's Ladies' Magazine, | 2.00 | 2.00 |
| Phrenological Journal, | 3.00 | 3.00 |
| Herald of Health, | 2.00 | 2.00 |
| Wood's Household Magazine, | 1.00 | 1.00 |
| "Little Corporal," | 1.50 | 1.50 |
| "Our Young Folks," | 2.00 | 2.00 |

Forward the price of any of the above publications to our address, and you will receive both journals regularly, for one year.

Address :—
THE BISTOURY,
ELMIRA, N. Y.

